# Autonomous Control of the Large-Angle Spacecraft Maneuvers in a Non-Cooperative Mission

**DOI:** 10.3390/s22228586

**Published:** 2022-11-08

**Authors:** Cheng Huang, Tianzeng Cao, Jinglin Huang

**Affiliations:** 1 Heilongjiang Provincial Key Laboratory of Complex Intelligent System and Integration, Harbin 150080, China; 2 School of Automation, Harbin University of Science and Technology, Harbin 150080, China

**Keywords:** non-cooperative target, attitude large-angle maneuver, attitude estimation, finite-time control, attention mechanism

## Abstract

Aiming at the large-angle maneuver control problem of tracking spacecraft attitude in non-cooperative target rendezvous and proximity tasks, under the condition that the target spacecraft attitude information is unknown and the actuator output has physical limitations, a limited-time autonomous control method is proposed. First, an end-to-end pose estimation network is designed based on adaptive dual-channel feature extraction and dual attention. The information around the target is obtained through the adaptive dual-channel feature extraction module. The addition of spatial attention and channel attention allows the network to learn the target’s characteristics more accurately. Secondly, based on the improved adaptive update law, a finite-time saturation controller is designed using the hyperbolic tangent function and the auxiliary system. The hyperbolic tangent function can strictly ensure that the control torque of the control system is bounded. Finally, the simulation results show that the proposed autonomous control method can accurately estimate the attitude of the non-cooperative target spacecraft and can maneuver to the target attitude within 20 s under the condition that the actuator’s output is physically limited.

## 1. Introduction

The large-angle maneuver control technology of spacecraft attitude has a wide range of applications in space missions, such as rendezvous and docking, on-orbit assembly, orbiting, and formation flying. It is one of the critical factors for completing various tasks. In recent years, finite-time control methods have been widely used to improve the accuracy and speed of spacecraft attitude control [1,2]. Compared with the cooperative situation, the rendezvous and proximity of non-cooperative targets have broader application prospects in space monitoring and service, deep space exploration, etc. However, it also presents more significant challenges due to the lack of information communication. Therefore, to meet the non-cooperative requirements of space missions, autonomous control modes that can identify non-cooperative target pose information and achieve attitude tracking are proposed [3,4].

At present, for pose estimation of non-cooperative targets, traditional methods rely on artificially designed features [5,6] and pose priors. The latter mainly establishes 2D-3D correspondence through feature point detection and matching and then obtains pose parameters by solving the Perspective-n-Points (PNP) [7]. However, in the face of complex lighting environments, soft textures, or rigid targets with complex structures, artificially designed features are insufficient in generalization and robustness. They are not suitable for the control method in this paper. To further improve the accuracy of attitude recognition, convolutional neural network technology [8] has received extensive attention and research, but it has not been widely used in spacecraft attitude estimation. Park et al. [9] use a vector to represent the target key point sequence and use EPnP (Estimation Perspective-n-Points) [10] to solve the target pose parameters; Chen et al. [11] use the HRNet (High-Resolution Net) [12] network to predict the particular vital points of the input image, and associate this point with the corresponding 3D model; The solution of attitude parameters adopts nonlinear optimization method. Sharma et al. [13] proposed the Spacecraft Pose Network (SPN) convolutional neural network to achieve 2D object detection. The relative pose of the target is estimated by using the image in the 2D detection frame, and the Gauss-Newton method is used to solve the translation under the constraints formed by the 2D detection frame and the relative pose.

For attitude controller design, in recent years, the mainstream design methods are mainly based on fixed time control and finite time control. When the initial information of the system is difficult to obtain accurately, there is a problem in that the convergence time cannot be calculated. To overcome this shortcoming, researchers propose a fixed-time control algorithm [14,15,16], which eliminates the dependence of convergence time on initial information and gives an upper bound of convergence time. Due to the characteristics of the fixed-time control method, it is mainly used in multi-agent systems. This allows the system to determine its own trajectory and the upper limit of the time to converge to the equilibrium point in advance through the adjustment of controller parameters under unknown initial conditions [17]. Finite-time control methods mainly include two categories: homogeneous theoretical methods and methods based on Lyapunov theory. Zou et al. [18] proposed a finite-time control method for spacecraft formation flight based on the homogeneous theory. Lu et al. [19] applied fast terminal sliding-mode control theory to finite-time control of spacecraft attitude tracking. Ran et al. [20] proposed a fault-tolerant finite-time attitude control method based on state observer using sliding-mode control theory. Further, Jiang et al. [21] proposed a finite-time output feedback attitude control method without angular velocity measurement by introducing the exponentiation integration technique. The rigid body spacecraft attitude system is a standard cascade system, and the backstepping method is a powerful design tool applied to the cascade system. Therefore, the finite-time control method based on the backstepping method has also been successfully applied to spacecraft attitude control. [22]. Although the finite-time controller described above enables the spacecraft attitude system to converge to an equilibrium point quickly and with high accuracy, it does not consider the actual physical limitations of the actuator. It is worth mentioning that, in order to guarantee a fast convergence rate, the finite-time control output always reaches a large magnitude, especially in the initial stage of the system response. Therefore, much literature has studied the finite-time saturation control method. Gui et al. [23] designed a bounded finite-time control method for attitude stabilization using homogeneous theory and saturation functions. By designing a finite-time observer and an adaptive controller, Zhang et al. [24] proposed a finite-time saturation control method for attitude tracking based on output feedback. Lu et al. [25] proposed a finite-time distributed cooperative attitude control method for multiple spacecraft considering input saturation by introducing Chebyshev neural network into fast terminal sliding mode. Considering the applicable background of the designed controller, we choose the finite time control method when the convolutional neural network can make the attitude control information a priori. So far, many finite-time control methods considering actuator saturation have been proposed. However, there are few research results on the use of backstepping. Moreover, although in [26,27], the backstepping method is used to design the spacecraft attitude control method that takes the actuator saturation into account, these controllers lack the necessary finite-time convergence proof, and the control process is discontinuous.

Therefore, this paper aims at the large-angle maneuvering control problem of tracking spacecraft attitude in non-cooperative missions, considering the accuracy and generalization requirements of the target attitude in space missions and the dynamic response speed of the finite time controller based on the backstepping method. An autonomous control method of attitude and large-angle maneuvering based on the combination of convolutional neural network and backstepping method is proposed in this paper. Compared with the existing literature, the contributions of this paper are:

- The adaptive dual-channel feature extraction module and the convolutional attention mechanism are integrated into the attitude estimation of the non-cooperative target spacecraft so that the network has higher accuracy and Robustness, which indirectly improves the adaptability of autonomous control;
- The participation of the backstepping method enables the finite-time saturation controller to effectively solve the input saturation problem even in the presence of external disturbances.

## 2. Spacecraft Attitude Control Model

The rotation matrix description method can avoid the singular problem of the Euler angle description method. Therefore, the kinematics and dynamics models of the spacecraft attitude described by the rotation matrix are as follows:

(1) $${\dot{R}}_{c}=R_{c}{\left(\omega_{c}\right)}^{\times }$$

(2) $$J_{c}{\dot{\omega }}_{c}+{\left(\omega_{c}\right)}^{\times }J_{c}\omega_{c}=u+d$$

(3) $${\left(\omega_{c}\right)}^{\times }=\left[\begin{array}{ccc} 0 & -\omega_{c3} & \omega_{c2}\\ \omega_{c3} & 0 & -\omega_{c1}\\ -\omega_{c2} & \omega_{c1} & 0 \end{array}\right]$$

$R_{c}\in SO\left(3\right)$ represents the rotation matrix of the spacecraft attitude from the body coordinate system to the inertial coordinate system; $\omega_{c}\in R^{3\times 1}$ is the representation of the spacecraft attitude angular velocity in the body coordinate system; $J_{c}\in R^{3\times 3}$ is the rotational inertia matrix of the spacecraft; $u\in R^{3\times 1}$ and $d\in R^{3\times 1}$ respectively represent the control torque of the spacecraft and disturbance torque.

$R_{e}$ and $\omega_{e}$ are the attitude and angular velocity errors, respectively, defined as $R_{e}=R_{t}^{Τ}R_{c}$ and $\omega_{e}=\omega_{c}-R_{e}^{T}\omega_{t}$, where $R_{t}$ and $\omega_{t}$ are the desired attitude and angular velocity, respectively.

The attitude error $R_{e}$ is a matrix that is difficult to apply to the design of the controller. This paper adopts another attitude error representation [24].

(4) $$e=\frac{1}{2\sqrt{1+tr\left(R_{e}\right)}}{\left(R_{e}-R_{e}^{T}\right)}^{\vee }$$

where $tr\left(R_{e}\right)$ represents the trace of $R_{e}$, and ∨ represents the inverse operation of the cross product.

According to ${\dot{R}}_{e}=R_{e}{\left(\omega_{e}\right)}^{\times }$, the spacecraft attitude tracking control model can be obtained as

(5) $$\dot{e}=E\omega_{e}$$

(6) $$J{\dot{\omega }}_{e}=F+u+d$$

(7) $$F=-{\left(\omega_{c}\right)}^{\times }J\omega_{c}-J{\left(\omega_{c}\right)}^{\times }\omega_{e}-JR_{e}^{T}{\dot{\omega }}_{t}$$

(8) $$E=\frac{1}{2\sqrt{1+tr\left(R_{e}\right)}}(tr\left(R_{e}^{T}\right)I_{3\times 3}-R_{e}^{T}+2ee^{T}$$

**Note 1.**  *For the rotation matrix $R_{e}\in SO\left(3\right)$,*$tr\left(R_{e}\right)$*is bounded and satisfies*$-1\leq tr\left(R_{e}\right)\leq 3$*; when $tr\left(R_{e}\right)=-1$*, *Equations (4) and (5) appear singular, so in order for the controller to operate normally, it is necessary to ensure that $-1\leq tr\left(R_{e}\right)\leq 3$is established, that is, to ensure the attitude error eis defined in set $L=\{R_{e}\in SO\left(3\right)\;|\;\|e\|<1\}$.*

## 3. Design of a Limited Time Autonomous Controller for Large Attitude Maneuvers

### 3.1. Pose Estimation Network Design

**Figure 1:**
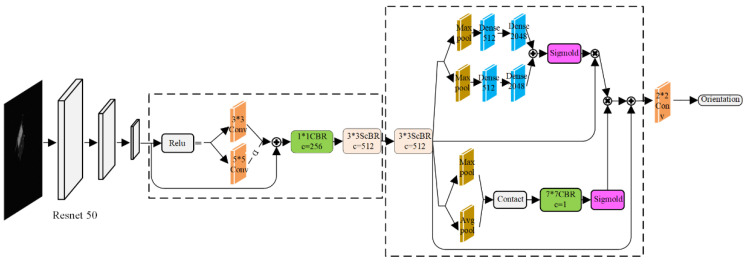
Network architecture.

This paper improves the URSONet [28] convolutional neural network and designs an end-to-end pose estimation network URSONet-Improve with the ResNet (Deep Residual Nework) as the backbone. The network architecture is shown in Figure 1.

As seen from the figure, after network URSONet-Improve removes the fully connected layer at the end of the original network, an adaptive dual-channel feature extraction module is added to reduce the loss of image feature information. Specifically, perform dual-channel feature extraction of 5×5 and 3×3 on the incoming features, and then add an adaptive coefficient α to the features extracted by the 5×5 convolution kernel. Then, the features extracted by the two channels are combined, and the 1×1 convolution is used to reduce the network parameters and integrate the information. Finally, the new features are extracted by the 3×3 depthwise separable convolution. This method can acquire features with different receptive fields and effectively combine the information around the target. At the same time, the residual structure is used to prevent gradient disappearance and gradient explosion.

The learned features are complex during network training, but not all are valid. Therefore, a convolutional attention module is introduced, which can increase the weight of useful features and weaken the weight of useless features during the training process to independently refine features in spatial and channel dimensions. Specifically, 3×3 depthwise separable convolution is used for feature extraction for the double-filtered features. The attention map is then inferred from two different dimensions, channel, and space, in turn. Then use, the spatial attention module and the channel attention module to divide the weights of the learned features for feature refinement. Finally, feature compression is done with a 2×2 convolution with a stride of 2.

### 3.2. Design of a Finite-Time Saturation Controller

In this paper, the idea of the backstepping method is adopted to design a limited-time saturation controller for spacecraft attitude maneuvering at large angles. Under the circumstance that the actuator output has physical limitations, an autonomous control mode based on the non-cooperative target attitude information output by the URSONet-Improve network is formed for tracking. The following are the relevant lemmas and assumptions for designing the controller.

**Lemma 1**  **[29].***Assuming that $\alpha_{1},\alpha_{2},\cdots ,\alpha_{n}$are all positive numbers, 0<ρ<2*, *then the inequality ${\left(\alpha_{1}^{2}+\cdots +\alpha_{n}^{2}\right)}^{\rho }\leq {\left(\alpha_{1}^{\rho }+\cdots +\alpha_{n}^{\rho }\right)}^{2}$holds.*

**Lemma 2**  **[30].** *Suppose the system* $\dot{x}=f\left(x\right),f\left(0\right)=0$ *has a continuously different* *iable function* V *, and the condition is satisfied:*

(1) *V is a positive definite function.*
(2) *There are positive real numbers* α>0 *, β>0, and γ∈(0,1), and an open neighborhood $U\subset U_{0}$ containing the origin, where $U\subset U_{0}$ holds $\dot{V}\leq -\alpha V-\beta V^{\gamma }$. Then the system is fast finite-time stable, and the convergence time T satisfies $T\leq \frac{1}{\alpha \left(1-\gamma \right)}ln\frac{\alpha V_{0}{}^{1-\gamma }+\beta }{\beta }$. If $U=U_{0}=R^{n}$* *, the system is globally fast and f* *inite-time stable.*

**Lemma 3**  **[19].** J *is a positive definite symmetric matrix, and* $\lambda_{min}$ *and* $\lambda_{max}$ *are the minima and maximum values in the eigenvalues of matrix* J *, respectively, for* $x\in R^{3\times 1}$ *,* $\lambda_{min}x^{T}x\leq x^{T}Jx\leq \lambda_{max}x^{T}x$ *holds.*

**Lemma 4**  **[31].** *For the system* $\dot{x}=f\left(x,u\right)$ *, suppose there is a continuously differentiable function* V *, and satisfy the condition:*

(1) *V is a positive definite function.*
(2) *There exists a positive real number* c>0 *and an open neighborhood* $U\subset U_{0}$ *containing the origin, where* $U\subset U_{0}$ *makes* $\dot{V}\leq -c$ *hold. Then the system is stable in finite time; if* $U=U_{0}=R^{n}$ *, the system is stable in global finite time.*

Assume that the external perturbation d is time-varying and bounded, $\|d\|\leq d_{max}$ and $d_{max}$ are known positive numbers, and both $\omega_{t}$ and ${\dot{\omega }}_{t}$ are bounded.

For the rigid body spacecraft attitude system (5)–(6), the following variables are introduced:

(9) $$x_{1}=e,x_{2}=\omega_{e}-\omega_{e}^{v}$$

According to the design idea of the backstepping method, and in order to ensure that the attitude error e is always defined in the set L, the virtual controller is selected as

(10) $$\omega_{e}^{v}=-\beta_{1}E^{-1}x_{1}-\beta_{2}E^{-1}f\left(x_{1}\right)+E^{-1}\lambda ln(1-x_{1}^{T}x_{1})x_{1}$$

(11) $$f\left(x_{1,i}\right)=\{\begin{array}{c} r_{1}x_{1,i}+r_{2}sign(x_{1,i})x_{1,i}^{2}\\ sig{\left(x_{1,i}\right)}^{\gamma }, \end{array}\;\;\begin{array}{c} \left|x_{1,i}\right|\leq \eta ,i=1,2,3\\ other \end{array}$$

In the formula, $sig{\left(x_{1,i}\right)}^{\gamma }=sign(x_{1,i}){\left|x_{1,i}\right|}^{\gamma },\;\;i=1,2,3$, $r_{1}=\left(2-\gamma \right)\eta^{\gamma -1}$, $r_{2}=\left(\gamma -1\right)\eta^{\gamma -}{}^{2}$, 0<γ<1, η are very small positive numbers, $\beta_{1}>0$, $\beta_{2}>0$.

**Proposition 1.**  *For Formula (9), when*$\omega_{t}$*and*${\dot{\omega }}_{t}$*are bounded, the virtual controller (10) can make*$x_{1,i}\left(i=1,2,3\right)$*converge to the region* $\left|x_{1,i}\right|\leq \eta$ *in a finite time.*

**Proof of Proposition 1.**  Choose Lyapunov function:

(12) $$V_{1,i}=\frac{1}{2}x_{1,i}^{2},i=1,2,3$$

Derive the function with respect to time to get ${\dot{V}}_{1,i}=x_{1,i}{\dot{x}}_{1,i}\;$, and substitute it into Equation (10) to get:

(13) $${\dot{V}}_{1,i}\leq -\beta_{1}x_{1,i}^{2}-\beta_{2}x_{1,i}f\left(x_{1,i}\right)$$

When $\left|x_{1,i}\right|\leq \eta ,i=1,2,3$, $x_{1,i}$ has converged to region $\left|x_{1,i}\right|\leq \eta$.

When $\left|x_{1,i}\right|>\eta ,i=1,2,3$, according to Lemma 1, combined with Formula (11), we can get:

(14) $${\dot{V}}_{1,i}\leq -\beta_{1}x_{1,i}^{2}-\beta_{2}x_{1,i}sig{\left(x_{1,i}\right)}^{\gamma }\leq -2\beta_{1}V_{1,i}-2^{\left(\gamma +1\right)/2}\beta_{2}V_{1,i}{}^{\left(\gamma +1\right)/2}$$

According to **Lemma 2**, $x_{1,i}$ can converge to the region $\left|x_{1,i}\right|\leq \eta$ in a finite time.

The upper bound square of the external disturbance is defined as the estimated quantity, and the continuous controller is designed based on the virtual controller (10) as follows:

(15) $$u=-F+J{\dot{\omega }}_{e}^{v}-E^{T}x_{1}-k_{1}x_{2}-u_{a}x_{2}$$

(16) $${\dot{\hat{\psi }}}_{i}=-\varepsilon_{1}{\hat{\psi }}_{i}+\frac{1}{2}p_{1}\chi^{-2}{\left|x_{2i}\right|}^{2}$$

In the formula, $u_{a}=diag(u_{\mathrm{ai}})$, $u_{\mathrm{ai}}=\left(\chi^{-2}{\hat{\psi }}_{i}\right)/2$, $\psi_{i}=d_{\mathrm{Mi}}^{2}$, $k_{1}>0$, $\varepsilon_{1}>0$, $p_{1}>0$, χ>0.

The hyperbolic tangent function is introduced to design a finite-time controller considering input saturation as follows:

(17) $$u=-k_{1}tanh(\varepsilon_{1}\varpi )-k_{2}tanh(\varepsilon_{2}x_{1})-k_{3}tanh(\varepsilon_{3}{\dot{x}}_{1})$$

To enable the system to converge to the equilibrium point in finite time, the auxiliary system is constructed as follows:

(18) $$\begin{array}{c} \dot{\varpi }=J^{-1}\left(F-J{\dot{\omega }}_{e}^{v}+u\right)+[x_{2}^{T}\left(F-J{\dot{\omega }}_{e}^{v}+u\right)+\\ x_{1}^{T}{\dot{x}}_{1}]\frac{\delta }{\delta^{T}\delta }+k_{4}\delta +\left(k_{5}+d_{max}\|x_{2}^{T}\|\right)\frac{\delta }{\delta^{T}\delta }+u_{a} \end{array}$$

(19) $$\delta =x_{2}-\varpi$$

(20) $$u_{a}=\{\begin{array}{cc} \frac{\delta }{\left\|\delta \right\|}\|J^{-1}\|d_{max} & \|\delta \|>n_{1}\\ \frac{sig{\left(\delta \right)}^{\gamma }}{n_{1}^{\gamma }}\|J^{-1}\|d_{max} & \|\delta \|\leq n_{1} \end{array}$$

In the formula, $k_{1}>0$, $k_{2}>0$, $k_{3}>0$, $k_{4}>0$, $k_{5}>0$, $\varepsilon_{1}>0$, $\varepsilon_{2}>0,\;\varepsilon_{3}>0,n_{1}>0$. □

**Theorem 1.**  *The spacecraft attitude systems (5)–(6), under the control of the finite-time controller (17) and the auxiliary system (18), can achieve the following goals:*

(1) *When $\left\|\delta \right\|>n_{1}$, variables $x_{1}$ and $x_{2}$ converge in finite time, and when $\left\|\delta \right\|\leq n_{1}$ and $k_{4}$ satisfies $k_{4}-\frac{\left(n_{1}^{\gamma }-{\left\|\delta \right\|}^{\gamma }\right)\|J^{-1}\|d_{max}}{n_{1}^{\gamma }\left\|\delta \right\|}\geq 0$, variables $x_{1}$ and $x_{2}$* *converge in finite time.*
(2) *The angular velocity error*$\omega_{e}$ *converges in a finite time.*

**Proof of Theorem 1.**  Choose the Lyapunov function:

(21) $$V_{3}=\frac{1}{2}x_{1}^{T}x_{1}+\frac{1}{2}x_{2}^{T}Jx_{2}+\frac{1}{2}\delta^{T}\delta$$

Derive it along the trajectory of the system (6), and substitute it into Equations (17) to (20) to get:

(22) $$\begin{array}{ll} {\dot{V}}_{3} & =x_{1}^{T}{\dot{x}}_{1}+x_{2}^{T}\left(F-J{\dot{\omega }}_{e}^{v}+u+d\right)+\\ & \;\delta^{T}\left[J^{-1}\left(F-J{\dot{\omega }}_{e}^{v}+u+d\right)-\dot{\omega }\right]\\ & =\delta^{T}J^{-1}d+x_{2}^{T}d-k_{4}\delta^{T}\delta -\left(k_{5}+d_{max}\|x_{2}^{T}\|\right)-\delta^{T}u_{a} \end{array}$$

When $\left\|\delta \right\|>n_{1}$,

(23) $$\begin{array}{ll} {\dot{V}}_{3} & \leq \|x_{2}^{T}\|d_{max}+\|\delta^{T}\|\|J^{-1}\|d_{max}-k_{4}\delta^{T}\delta -\\ & k_{5}-\|x_{2}^{T}\|d_{max}-\|\delta \|\|J^{-1}\|d_{max}\\ & =-k_{4}\delta^{T}\delta -k_{5}\\ & \leq -k_{5} \end{array}$$

When $\|\delta \|\leq n_{1}$ and $k_{4}$ satisfies $k_{4}-\frac{\left(n_{1}^{\gamma }-{\left\|\delta \right\|}^{\gamma }\right)\|J^{-1}\|d_{max}}{n_{1}^{\gamma }\|\delta \|}\geq 0$,

(24) $$\begin{array}{ll} {\dot{V}}_{3} & \leq -k_{4}\delta^{T}\delta -k_{5}+\|\delta^{T}\|\|J^{-1}\|d_{max}-\frac{{\left\|\delta \right\|}^{\gamma +1}\|J^{-1}\|d_{max}}{n_{1}^{\gamma }}\\ & =-k_{5}-k_{4}\delta^{T}\delta +\left(1-\frac{{\left\|\delta \right\|}^{\gamma }}{n_{1}^{\gamma }}\right)\|\delta \|\left\|J^{-1}\right\|d_{max}\\ & =-k_{5}-\left[k_{4}-\frac{\left(n_{1}^{\gamma }-{\left\|\delta \right\|}^{\gamma }\right)\|J^{-1}\|d_{max}}{n_{1}^{\gamma }\|\delta \|}\right]\|\delta {\|}^{2}\\ & \leq -k_{5} \end{array}$$

According to **Lemma 4**, the variables $x_{1}$ and $x_{2}$ converge in finite time. (1) is proved.

According to the form of Equations (9) and (10), combined with the finite time convergence of $x_{1}$ and $x_{2}$, it can be concluded that the angular velocity error $\omega_{e}$ converges in finite time. (2) is proved.

To sum up, **Theorem 1** is proved. □

**Note 2.**  *The finite-time controller designed in this paper is continuous, and there is no chattering phenomenon. The adaptive update law (16) will not always be positive, avoiding the phenomenon that the estimated value may increase indefinitely so that the control torque in the steady state stage will not be too large.*

## 4. Simulation Verification

### 4.1. Pose Estimation

In order to verify the performance of the network proposed in this paper, this paper selects the dataset in the “Pose Recognition Challenge” jointly organized by the European Space Agency (ESA) and Stanford University (SLAB) for experimentation. The dataset provides Tango satellite images with a pixel size of 1920×1200. Table 1 gives the number of real and simulated images in the training and test sets.

The loss function is defined as follows:

(25) $$Loss=\beta L_{ori}$$

(26) $$L_{ori}={\left|1-\overset{m}{\underset{i=1}{\sum }}y^{\left(i\right)}*y_{ot}^{\left(i\right)}\right|}^{2}$$

where β is the fine-tuning loss weight, $y^{\left(i\right)}$ and $y_{ot}^{\left(i\right)}$ are the estimated pose value and the true pose value conversion vector, respectively.

**Figure 2:**
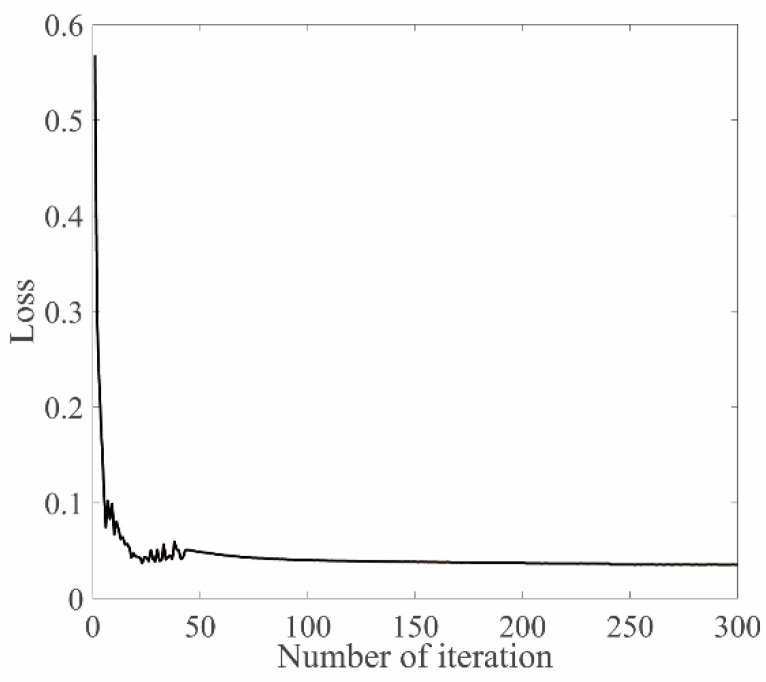
Training curve.

The training adopts the SGD (Stochastic Gradient Descent) optimizer; the training batchsize is 256, the initial learning rate is 0.0001, and the weight decay coefficient is 0.001. Figure 2 shows the change in the loss value of the training set during the training process of the network in this paper. It can be seen from the figure that the loss value of the training set decreases steadily and gradually converges, indicating that the model has stability and convergence.

**Figure 3:**
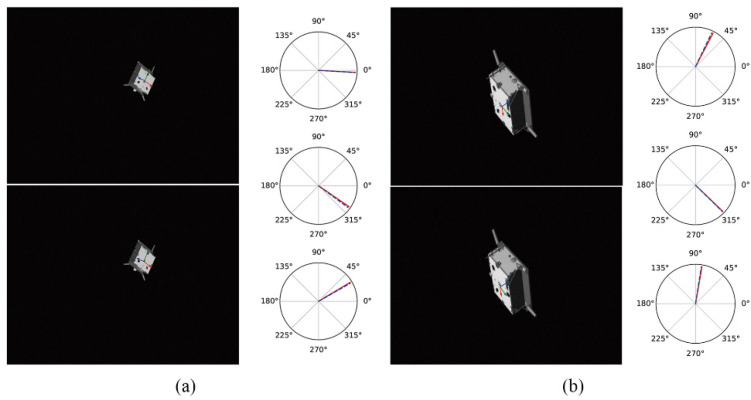
Attitude estimation results of non-cooperative spacecraft in a single background. (**a**) is the estimation result at a long distance under a single background (**b**) is the estimation result at close range under a single background.

**Figure 4:**
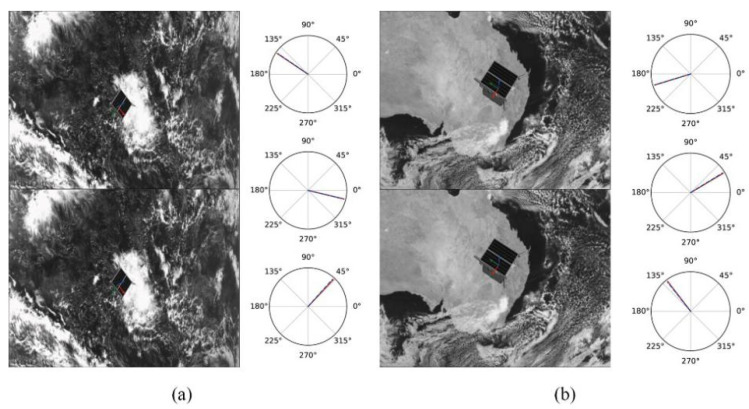
Attitude estimation results of non-cooperative spacecraft in complex background. (**a**) is the estimation result at a long distance under a complex background (**b**) is the estimation result at close range under a complex background.

Table 2 shows the experimental results of different models under the same data set. The estimated results are uploaded to the KPEC (Kelvins Pose Estimation Challenge Copetition) platform, and the test set scores are obtained from the online evaluation. The advantages and disadvantages of this method are analyzed by comparing it with other methods of the same type. $E_{syn}$ and $E_{real}$ represent the simulated image score and the real image score, respectively; URSONet-D(3×3) represents only two 3×3 adaptive dual-channel feature extraction for the incoming features; URSONet-D(5×5) represents 3×3 and 5×5 adaptive dual-channel feature extraction for incoming features; URSONet-S represents adding spatial attention mechanism based on URSONet-D; URSONet-C represents adding channel attention mechanism based on URSONet-D; Top 10 average is the average score of the top 10 in the “Pose Recognition Challenge”. Analyzing Table 2, it can be concluded that the addition of the adaptive dual-channel feature extraction module improves the accuracy of the network output attitude angle error by 6.2%, and when the adaptive dual-channel feature extraction module and the convolutional attention module act at the same time, the accuracy of the network output attitude angle error is improved, and the improvement rate is 18.1%. Figure 3 and Figure 4 show the comparison of the network predictions with the ground truth. Figure 3 and Figure 4 represent attitude estimation results in a single deep space environment and in a complex background, respectively. In each sub-figure in the figure, the left side represents the results of the spacecraft’s real attitude and predicted attitude in the form of coordinate axes, and the right side of the figure represents the true value and predicted value in the form of Euler angles. The sub-figure on the right is the roll angle, pitch angle, and yaw angle from top to bottom, with the solid red line being the true value of the attitude and the blue dotted line being the predicted value of the attitude. It can be seen from the figure that the position and pose prediction values estimated by the method proposed in this paper have almost no deviation from the real value. The experimental results verify the accuracy and rationality of the pose estimation network proposed in this paper.

### 4.2. Attitude Tracking

In order to verify the effectiveness of the proposed controller, the relevant numerical simulations are carried out in this section as follows; the tracking spacecraft tracks the target spacecraft, and its moment of inertia and initial motion state are:

(27) $$J=\left[\begin{array}{ccc} 27.7 & 0.3 & -0.2\\ 0.3 & 23.5 & 0.5\\ -0.2 & 0.5 & 24.6 \end{array}\right]\mathrm{kg}\cdot m^{2}$$

(28) $$R_{c}\left(0\right)=\left[\begin{array}{ccc} -0.5414 & -0.7072 & -0.4546\\ -0.0009 & -0.5403 & 0.8415\\ -0.8407 & 0.4560 & 0.2919 \end{array}\right]$$

(29) $$\omega_{c}\left(0\right)={\left[\begin{array}{ccc} 0.1 & 0.1 & 0.1 \end{array}\right]}^{T}\;\mathrm{rad}/s$$

The spacecraft attitude obtained by the attitude estimation network is shown in Figure 3a.

(30) $$\theta_{t}={\left[\begin{array}{ccc} -3.0894125 & -34.4091333 & 31.022785 \end{array}\right]}^{T}$$

(31) $$R_{t}=\left[\begin{array}{ccc} cos\delta_{t}cos\theta_{t} & sin\phi_{t}sin\delta_{t}cos\theta_{t}-cos\phi_{t}sin\theta_{t} & cos\phi_{t}sin\delta_{t}cos\theta_{t}+sin\phi_{t}sin\theta_{t}\\ cos\delta_{t}sin\theta_{t} & sin\phi_{t}sin\delta_{t}cos\theta_{t}+cos\phi_{t}sin\theta_{t} & cos\phi_{t}sin\delta_{t}cos\theta_{t}-sin\phi_{t}sin\theta_{t}\\ -sin\delta_{t} & sin\phi_{t}cos\delta_{t} & cos\phi_{t}cos\delta_{t} \end{array}\right]$$

In (31), θ is defined as the yaw angle, δ is the pitch angle, and ϕ is the roll angle. The rotation matrix of the target spacecraft can be calculated as:

(32) $$R_{t}=\left[\begin{array}{ccc} 0.7070141 & -0.4885306 & -0.5113404\\ 0.4251997 & \;0.8714131 & -0.2446312\\ 0.5650985 & -0.0444641 & 0.8238244 \end{array}\right]$$

The disturbance torque is $d=0.002{\left[\begin{array}{ccc} sin(0.1t) & cos(0.2t) & sin(0.2t) \end{array}\right]}^{T}N\cdot m$.

**Figure 5:**
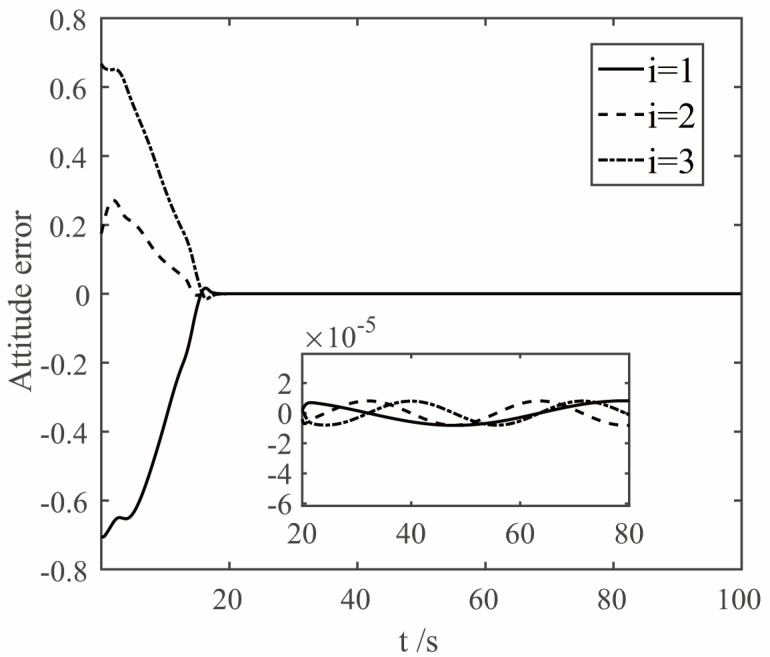
The curves of attitude error.

**Figure 6:**
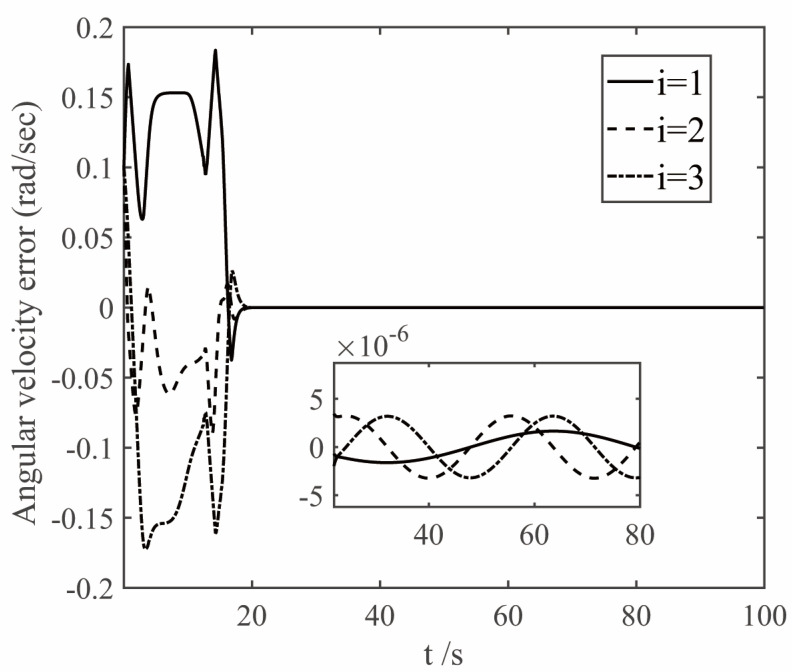
The curves of angular velocity error.

**Figure 7:**
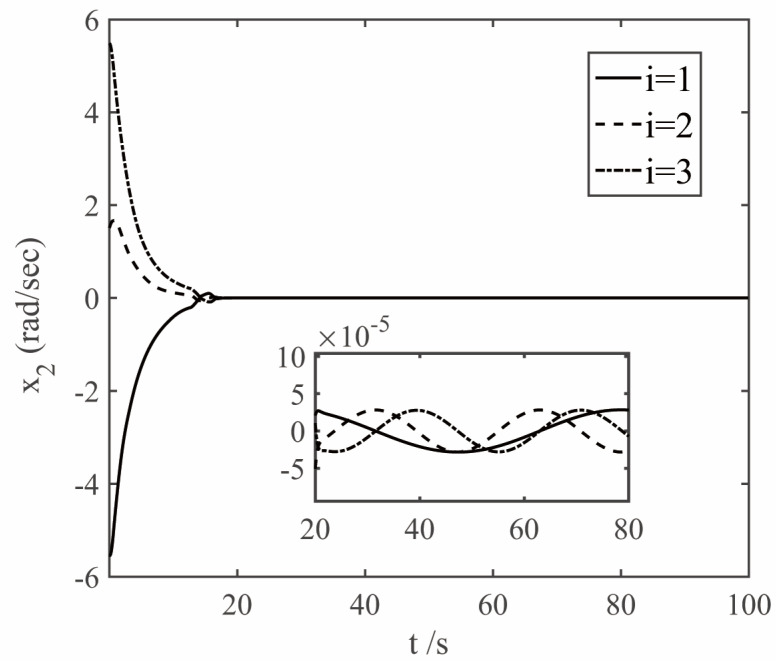
Curves of *x*_2_.

**Figure 8:**
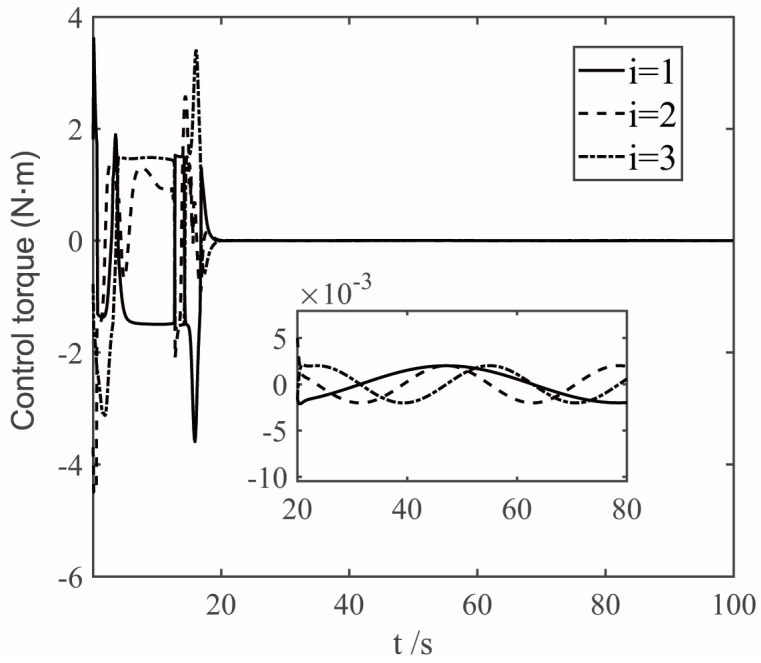
The curves of control torque.

The parameters of the finite time controller (17) and the auxiliary system (18) are selected as $k_{1}=1.5$, $k_{2}=1.5$, $k_{3}=1.5$, $k_{4}=7$, $k_{5}=0.002$, $\varepsilon_{1}=50$, $\varepsilon_{2}=50$, $\varepsilon_{3}=50$, $\beta_{1}=0.3$, $\beta_{2}=0.3$, γ=0.8, η=0.001, $n_{1}=0.001$, λ=0.001. In order to deal with the chattering problem of $\frac{\delta }{\delta^{T}\delta }$ when δ is close to 0 [23], $\delta^{T}\delta +0.001$ is used to replace $\frac{\delta }{\delta^{T}\delta }$ when $\delta^{T}\delta \leq 0.001$. The simulation results are shown in Figure 5, Figure 6, Figure 7 and Figure 8. The figure represents the $i^{th}$ element of the vector, and the variable $x_{2}$ is defined in Equation (9), which is the deviation between the angular velocity error and the virtual control command.

Analysis of Figure 5, Figure 6 and Figure 7 shows that under the control of the finite-time controller (17), the errors of the pitch, yaw and roll angles of the system increase in the initial stage. After the error reaches the maximum at about 3 s, it decreases rapidly, and finally gradually converges to 0. It can be seen from the partial enlarged diagram that the steady-state error of the controller here is very small, and the error value of the proposed finite-time controller is much lower than that of the conventional controller. And the attitude error and angular velocity error converge to the area near zero with high precision within 20 s, and the controller can make the tracking spacecraft track the target spacecraft.

Figure 8 shows the simulation curve of the control torque of the tracking spacecraft. The control torque curve shown in Figure 8 shows that the output torque at the initial stage of control is small, and the actual output torque in the entire control process satisfies the physical constraint u≤5 N·m. It can be seen from the curve that the controller ensures the continuity and effectively eliminates the chattering phenomenon. At the same time, it ensures cancellation of the permanent disturbances d.

## 5. Conclusions

This paper focuses on the problem of limited-time autonomous control of spacecraft attitude and large-angle maneuvering in non-cooperative target rendezvous and rendezvous and proximity tasks and innovatively combines a convolutional neural network with backstepping-based limited-time saturation control for this problem. The designed controllers can achieve finite-time convergence of the closed-loop system and avoid chattering. The simulation results show that the proposed control method can ensure that during the rendezvous process, the spacecraft attitude angle can not only quickly converge to the desired attitude, but also ensure good control accuracy. In our future research, we hope to consider some other issues based on the current research, such as: considering the situation without angular velocity feedback, by designing a finite-time observer to develop a finite-time saturation control scheme for attitude tracking based on output feedback.

## Figures and Tables

**Table 1:** Number of Dataset.

	Real Images	Simulated Images
Training set	5	12,000
Test set	300	2998

**Table 2:** Comparison of experimental results.

Model	$E_{syn}$	$E_{real}$	$e_{q}\left({}^{{}^{\circ}}\right)\;$
URSONet	0.0604	0.1630	5.46
URSONet-D(3×3)	0.0531	0.1561	5.35
URSONet-D(5×3)	0.0462	0.1464	5.12
URSONet-S	0.0442	0.1448	5.13
URSONet-C	0.0424	0.1430	4.88
URSONet-Improve	0.0296	0.1328	4.74
Top 10 average	1.3848	0.1515	10.82

## Data Availability

The data presented in this study can be made available on request from the corresponding author. The data are not publicly available due to confidentiality agreements, supporting data can only be made available to bona fide researchers subject to a non-disclosure agreement.
